# Factors Affecting the Effectiveness of Hospital Incident Command System; Findings from a Systematic Review

**DOI:** 10.30476/BEAT.2020.46445

**Published:** 2020-04

**Authors:** Paria Bahrami, Ali Ardalan, Amir Nejati, Abbas Ostadtaghizadeh, Arezoo Yari

**Affiliations:** 1 *Department of Health in Emergencies and Disasters, School of Public Health, Tehran University of Medical Sciences, Tehran, Iran*; 2 *Pre-Hospital and Hospital Emergency Research Center, Tehran University of Medical Sciences, Tehran, Iran*; 3 *Social Determinants of Health Research Center, Research Institute for Health Development, Kurdistan University of Medical Sciences, Sanandaj, Iran*

**Keywords:** Hospital, Incident Command System, Assessment, Effectiveness

## Abstract

**Objective:**

To examine all aspects affecting the functioning of the system and the most important factors in its assessment through a systematic review during 1990 to 2017.

**Methods:**

This systematic review of the current literature study was conducted during July 2017, and all articles, books, guidelines, manuals and dissertations pertaining to the Incident Command System were analyzed. A total of articles and relevant documents were identified and finally these articles, which we found, were analyzed based on the specified indicators.

**Results:**

In this research 992 articles and relevant documents were identified and eventually, 48 articles were included and analyzed. The results were categorized into 6 main groups including 65 subgroups and 221 variables: features of hospital incident command system (14 subgroups and 53 variables), strengths of the system (15 subgroups and 70 variables), weaknesses of the system (10 subgroups and 15 variables), factors influencing the system's performance improvement (12 subgroups and 42 variables), factors that reduce the effectiveness of system include 11 subgroups (10 internal factors and 1 external factor) and 22 variables and important factors in assessing system performance (2 sub-groups and 19 variables).

**Conclusion:**

According to the results, Evaluating the effectiveness of a hospital accident command system (HICS) in a valid method can improve the efficiency of this system. In this appraisal, hospital managers and health decision-makers should consider principles, characteristics, strengths and weakness of it.

## Introduction

Today, the occurrence of unexpected incidents around the globe affects governments and nations causing a great number of fatalities and significant economic losses. Despite many recent advances, incidents and disasters remain one of the most important concerns of a man's life [1]. Therefore, it is important to pay more attention to the development of plans, principles on natural disaster reduction and their effects, as well as the proper management of disaster risk reduction [2]. Among the many components involved in crisis management, relief and rescue in emergencies, healthcare centers, especially hospitals, play a major role [3]. Due to the lack of predetermined structures for proper management and focus on activities and training programs, these healthcare centers may expose to multiple risks and performance dysfunction [4]. Therefore, hospital preparedness is the main element of disaster management programs, which should be based on a standard protocol [5]. One of the invaluable management instruments which has an effective role in empowering services of healthcare centers according to global experiences is the Hospital Incident Command system (HICS) [6]. Hospital Incident Command system is a management system used to help manage incidents in unexpected situations and makes an attempt to build a coordination between hospitals and other institutions involved through using a rational and integrated management structure, responsibilities and duty description, creation of transparent reporting channels, and building a simplified and commonplace terminology system [4,7]. Regardless of their size or ability to provide care for the patient, health centers, especially hospitals, can use the system for planning and responding at all*-*hazard emergency situations [8]. According to the reports published by the organizations in the United States, the system has been able to improve the quality of delivered hospital services in crisis situations very effectively and offered many effective crisis management plan and strategies for the hospital [4]. HICS was designed in 1991 as one of the strategies of hospitals for contrast with disasters with the mission of prevention, mitigation, response, and recovery in hazards [6]. The Hospital Incident Command System (HICS) is widely used by hospitals, yet there is a paucity of research and a lack of developed models to examine HICS implementation [6 ,9]. In addition, our review shows that there has been no comprehensive study analyzing the positive or negative aspects of the system [9]. Perhaps the importance to develop HICS for emergencies has led scholars to ignore its qualitative aspects [6]. Therefore, in order to improve the effectiveness of Hospital Incident Command System, the current study aimed to assess the effectiveness of system with a focus on its features, strengths, weaknesses, factors contributing to the increasing and decreasing of system effectiveness, as well as important factors involved in system assessment. The aim of the current study was investigating and categorize factors affecting HICS effectiveness with a focus on its features, strengths, weaknesses, factors contributing to the increasing and decreasing of system effectiveness, as well as important factors involved in system assessment to improve the effectiveness of HICS. Therefore, the findings of this study can increase the knowledge of decision makers about the HICS in order to improve hospital readiness and respond appropriately during accidents and disasters.

## Materials and Methods

*Data source*

A systematic review was conducted to investigate published studies and documents relating to the factors affecting the performance of Incident Command System.

This research was conducted during July 2017.All articles, books, guidelines, manuals and related dissertations were extracted from January 1, 1990 to July 15, 2017.   We searched databases including PubMed, Ovid, Springer, Scopus, science of the Web and Google Scholar, Medline for foreign articles, and Iran's Medicine and SID for Persian articles. In addition, we searched ProQuest databases for relevant dissertations. Relevant articles were identified by searching citations and using the snowball mechanism.

*Search Strategy*

Other than the articles searched using the snowball method, the rest of the articles included in the study was searched using Medical Subject Headings (MeSH) *resource*: ICS OR "Incident Command Systems" OR "hospital incident command system" OR "hospital emergency incident command system "OR" Incident Command Structure "OR" Incident management framework "AND utilization OR evaluation OR effectiveness OR" Measures of effectiveness "OR performance OR implementation OR assessment OR application OR ORGANIZATION or strengths OR weak points AND" Emergency Operation Center "OR out -of hospital "OR" EOC "OR" Pre hospital "" hospital "OR" Emergency medical system ".

*Inclusion criteria*

The inclusion criteria were: articles published in the scientific journals and were relevant to research questions, published in English language and only allocated to Hospital Incident Command System in hospital and pre-hospital settings, Hospital Emergency Operation Center (HEOC) and health centers.

*Exclusion criteria*

**Fig. 1:**
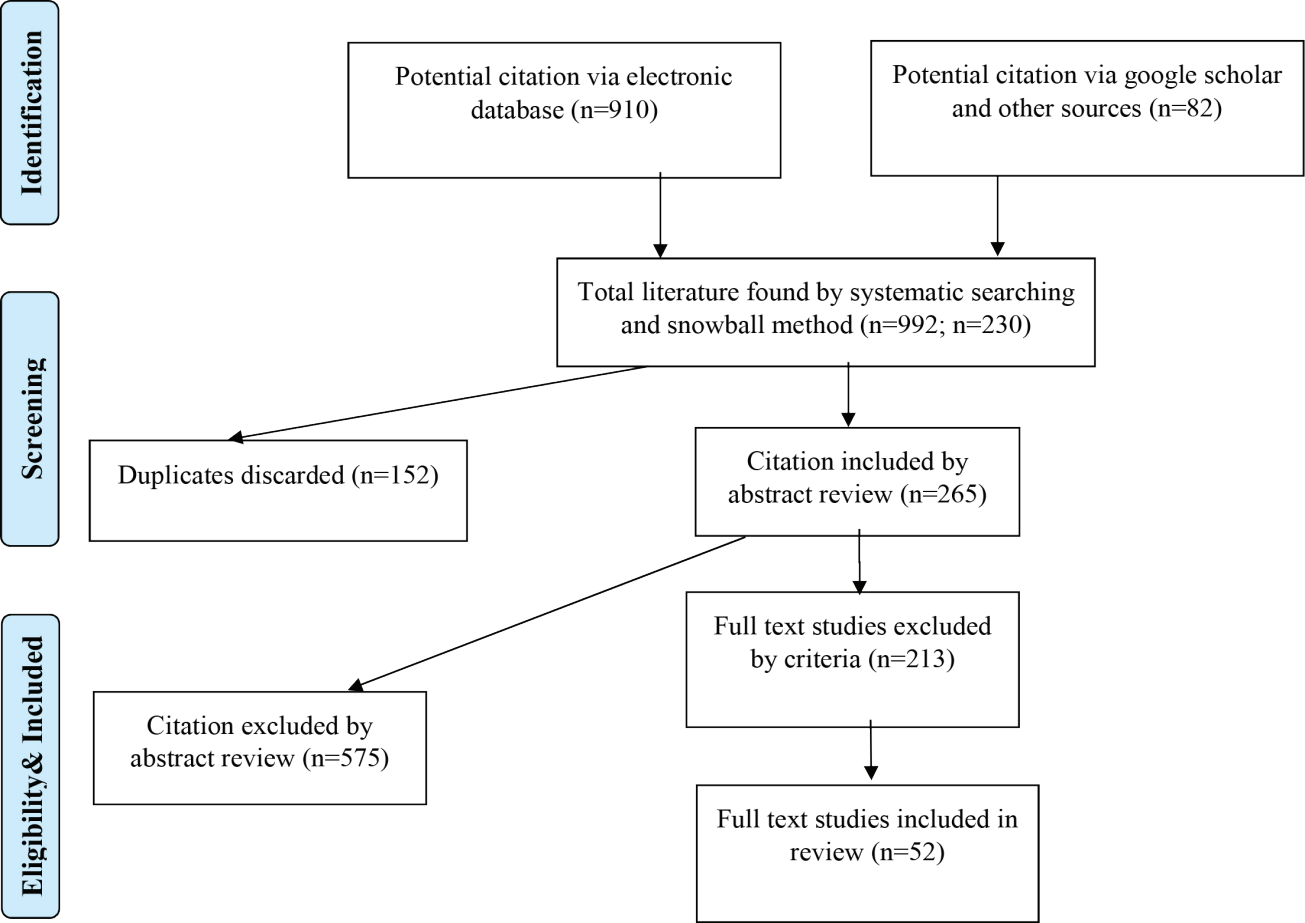
Flow diagram of the search and selection of papers

The exclusion criteria were as follows: published papers before 1990, published in non-academic journals, not relevant to research questions, not in English and duplicate publications. Further, articles and texts were analyzed using descriptive and thematic approaches. Quality assessment of included studies was done via PRISMA checklist. Figure 1 presents a flow diagram of data collection and its analysis. The included studies in the current systematic review are summarized in Table 1 and the categorization of the included articles based on their strengths and subjects is demonstrated in Table 2. 

*Quality assessment*

A 7-question checklist was produced to assess the quality of the retrieved publications by authors (Table 3). The Quality-related questions investigated the following components:1- Number of subgroups mentioned in the main group of hospital incident command system features 2- Number of subgroups mentioned in the main group of strengths of the system, 3- Number of subgroups mentioned in the main group of weaknesses of the system, 4- Number of subgroups mentioned in the main group of factors influencing the system's performance improvement, 5- Number of subgroups mentioned in the main group of factors that reduce the effectiveness of system 6- Number of subgroups mentioned in the main group of variables and important factors in assessing system performance 7- The number of main groups referenced.

*Thematic analysis*

In the first step, one researcher analyzed the extracted data of each publication by thematic method (PB), In the second phase, the draft analysis was reviewed by the second researcher (AY), this appraisal continued until two researchers had agreed on themes and sub-themes was attained. Any disagreement or mismatch was resolved through discussion and involvement a third researcher (AOT).

## Results

A total of 992 articles and documents were searched and finally 52 articles were analyzed based on the specified indicators. The data were analyzed descriptively. Most of the reported citations were related to the United States (59.6), Iran (19.2), China (3.8), Saudi Arabia (3.8) and Australia (3.8). About 92.26% of the articles included in the study published from 2014 to 2017 and 9.51% from 2006 until the end of 2013, concurrent to the fifth and fourth editions, respectively, by the Hospital Incident Command System. An approximately 23% of the articles used either absolute or qualitative approaches and 5.11% were case studies. In addition, the focus of the selected articles ranged from the application of the system in the hospital or pre-hospital system (4.63%), health centers (3.17%), both hospital and health centers (6.7%) and Hospital Emergency Operation Center (HEOC) (7. 5%).Therefore, according to the results, the hospital and pre-hospital settings had the highest rate of system referrals. The abstracts of the relevant articles and documents are shown in Table 1.

*Thematic analysis*

The results were categorized into 6 main groups, 65 subgroups and 221 variables as follows: features of hospital incident command system (14 subgroups and 53 variables), strengths of the system (15 subgroups and 70 variables), weaknesses of the system ( 10 subgroups and 15 variables), factors influencing the system's performance improvement (12 subgroups and 42 variables), factors that reduce the effectiveness of system include 11 subgroups (10 internal factors and 1 external factor) and 22 variables and important factors in assessing system performance (2 sub-groups and 19 variables).

*Principles and features effective in the success of the hospital emergency hospital system:*

The hospital incident command system is a management system for controlling, commanding and coordinating the activities of independent groups. This system is designed to achieve the common goal of incident prevention, reduction of mortality, financial losses and severe damage. The system has several features that contribute to achieving these goals and affect its success. The present study indicated that there are several significant features affecting the effectiveness of hospital incident command system including the organized command structure [10] based on the same principles and compliance with the key structure of the incident command [9,11,12] use of a bureaucratic framework based on military principles [13]. This system is characterized by an organizational form of the structure [6], hierarchical* structure* [13-15], and a specific command chain [16]. In addition, this system provides an appropriate and reliable structure for leadership regardless of the incident type [17]. A clear spoken language —based on the common [4,6,9-12, 16, 18], simple and commonplace [4,18] terminology is another feature of the system.the hospital incident command system can define specific  organizational functions and roles through job descriptions [11, 19,20], rational tenets for the allocation and organization of occupational activities [14] clarify the precise managerial tasks and responsibilities [21], access to a list of personal job descriptions [22], positions [4, 18], responsibilities [4, 18, 20, 23, 24], individuals duties [4, 7, 16, 18, 23, 25- 28], external organizations [28], responsibilities of hospital incident command system team [29],Specifying specific roles in an organizational table [22,23], and consequently prioritize tasks based on the job description worksheets [6] and recruit the personnel when it becomes necessary [30]. Additionally, numerous studies have focused on other structural features of Hospital Incident Command System including modularity [12], flexibility [13-16, 19, 23,31], both flexibility and modularity [4, 6-8, 11, 14, 18, 32], analogy and comparison [9], compatibility [17], adaptability in crisis situations at a variety of scales [17,18, 23], and category [26,31] and concordance with planned and unplanned events [33] and effective for management in potentially changing environments [10]. The hospital incident command system is based on the principles and characteristics mentioned. Applying these principles and features will ensure the utilization of resources and reduce policy discrepancies and the operations of accountable organizations.

*Strengths of system use*

Setting up a precise, efficient and cost-effective managment system is one of the main pillars of disaster management programs in hospitals. The hospital incidnet command system brings significant benefits to hospitals and plays an important role in improving the quality and delivery of services during emergencies and disasters in hospitals. Hospital incident command system provides a powerful framework [10] standard template [8], structured and organized incident response [16] and facilitates emergency incident response [19]. various advantages for hospital incident command system including provision of the most reliable management protocols [18], and comprehensive crisis management strategy [8] can apply to improve the management capabilities of the emergency [31]. HICS develops strategies for effective and efficient dealing with crisis situations [4], accelerates effective and quick response [8,11], increases effectiveness [18, 23], enhances efficiency and effectiveness of the response plan [31, 34], facilitates communication between different units of the system in emergency incidents [35], fosters problem solving process among the organizations involved [22,25] and ultimately, reduces the chance of errors and parallel work [25].

Hospital incident command system provides a response planning [36] this system can offer hospital of all sizes with an opportunity to plan, prepare and respond to both emergency and non-emergency situations, make other relevant units  and organizations take part in the emergency response depending upon the size and type of incident  [4, 18], extend or limit the size, scope*,* and complexity of an incident*,* assign particular tasks or position based on the magnitude of the incident [8] accelerates effective and quick response [8,11] and consequently guarantees the successful implementation of the plan [37]. Furthermore, numerous studies have mentioned another strength of the system including: detection of system capabilities by other organizations [8], providing a system for coordination [12] coordination between hospitals [4, 23, 38] and accountable organizations [4,6-8, 23], coordinating activities among independent groups and coordinated response actions [6] coordinated response to emergencies situations [8, 11, 35, 39], coordinated multi-disciplinary response to public health threats [28], mutual efforts [40], decentralization in decision-making [10] and building unity in dealing with complex and extreme crises, making incident command system known to the public [7].

Additionally another advantages for hospital incident command system including improvement of administrative communications [8, 9], developing a system [12] and communication plan [6], quick and easy communication with other crisis management systems in various rescue and relief departments [4], and facilitated communications [16, 24] between hospitals , Medical emergencies and other responsible organizations [12] and foreign organizations [10], clear inter-organizational communication [39], inter-team environmental awareness [41], avoidance of unnecessary communications [26], improved communication [42, 43], and existence of effective communication plans [6]. Hospital incident command system with some its features can lead to the cost minimization [4, 18, 22, 23]: provision of accurate and timely documentation on spending and resource utilization, [11] reduced financial losses and severe injuries, [6] effective use of resources, [22] provision of adequate and efficient medical facilities and personnel [39], effective use of all resources for problem solving [17], provision of resources and equipment [30,44], provision of health care facilities for emergency management [33], use of manpower on a regular basis [45], providing health facilities needed for emergency management [33], regular staffing [45] and sharing resources in organizations and health care centers [6, 11]. So considering the strengths of this system, HICS has been identified as one of the factors necessary to boost resilience [42], hospital empowerment [6], incident management and reduced fatalities [6], response to daily operations and emergency and non-emergency situations [22].

*Weakness of system use*

according to reports, since the system was initially developed for use in a military and hierarchical structure, it makes hard for the providers of public health system to use the system because of cultural differences [19]. Buck DA and colleagues also stated in their study that although the system was successful in firefighting organizations, it has not been successful in some organizations, such as public health [45]. Similarly, SA Andrew *et al*. questioned the usefulness capabilities of system in reducing organizational disputes, especially at large-scale incidents and events [46] and FM Burkle *et al*. found that the system is unable to manage the complexities of a large*-*scale health*-*related disaster*, *especially epidemic situations [47]. the main disadvantage is that the structure cannot be changed or be reliable in terms of training, coordination and administrative capability [48].Furthermore, Timm NL *et al*. reported that hospital staff were completely unfamiliar with the language system and were unable to use terms such as logistics, operations, financial to explain leadership roles [48].The vastness of the range of job descriptions in the system is another weakness of the system [48] Likewise, NL Timm *et al*. noted the inefficiency of the system in real time response and exposure to stress [48]. Some studies have also referred to high cost as one of the other problems in the system [23]. R Rimstad’s findings suggesting that system sharing between partner organizations is a major problem [13]. 

*Factors affecting in increasing efficiency*

Considering the importance of the incident command system in managing and responding effectively to emergencies and disasters, and given the widespread use of this system in hospitals in the world, it is essential to pay attention to factors that increase its efficiency. Therefore, it is important to pay special attention to several factors for increasing the effectiveness of the system within the hospitals, including familiarity with the organizational structure of the system [18], creation of cluster structures for the system at the planning stage depending on the response phase [47] determining the organizational hierarchy of the structure based on the requirements rather than the titles [17] development of units, positions, description of new duties in accordance with hospital requirements [14] definition of job description and supervision of managers [22] and providing training and information to managers at all levels for creation of a common language and building culture [23].

The application of advanced communication technology to coordinate and provide critical information between incident command teams [41] has been one of the most important elements in improving system performance, furthermore It is necessary to pay special attention to regional coordination plays a significant role in the promotion of system efficiency [49]. Also the existence of transparent reporting channels [7, 20, 23-25] and transmission of information to high-level authorities [10] are the two main aspects of the incident command system, which can facilitate the information collection, reporting [28], sharing information [31], information acquisition and information dissemination [50]. In order to improve the effectiveness of the system, some additional measures need to be taken: removing financial barriers to implement the system, [23] financing the hospital to establish the system, [23] providing procurement* opportunities* for staff and offices to strengthen the crisis management culture [15], supporting the Ministry of Health and taking into account additional funding for promotion of system [22].compliance with the rules and regulations [23], compliance with instructions and guidelines among the personnel and medical staff [37], enhancing compliance with the system principles [35] developing guidelines and regulations for hospitals and legal requirements [22].

Taken together, improving the effectiveness of the Hospital Incident Command System depends on understanding and recognizing the features and principles of the system [12, 18, 23, 37], training employees in order to understand the system [9, 23,50],  holding continues specialized training courses [9], implementing exercises and procedures [10,24,41], building an administrative commitment and support for managers [9], creating an interest in the personnel [22], matching the system to the needs, updating and implementing the native version of the system [4, 49] eliminating financial barriers [22, 23] and complex and challenging administrative obstacles [22], appointment of competent, experienced and qualified managers [13, 22] and understanding the strengths and weaknesses of the system. 

*Factors decreasing system efficiency*

In contrast to the above finding, Timm NL *et al*. showed that while implementing the system, the conflicts from confused job responsibilities and roles can lead to inefficiencies in response and real implementation of the system may be failed [48] and the incompatibility of this system with the management structure of hospitals can decrease the system's effectiveness [49]. YarMohammadinia *et al*. found that the lack of legal requirements with continuous regulatory change, and the absence of unity of command [23], can weaken and decrease the system's efficiency and effectiveness [23]. Furthermore, the lack of a general method for assessing HICS and hospital-based exercise programs [25], lack of cultural management for crisis command, lack of the need to create this system by managers, lack of support and commitment from authorities and managers, shortage of qualified managers at all levels, paying attention to daily activities by managers, lack of a common management language [23] and lack of senior management commitment to system implementation of the [9] can attenuate system performance.

*Points to be considered in the organization's assessment*

Assessing the hospital incident management system leads to identifying the weaknesses, strengths, decreasing factors and increasing the efficiency of the system. By identifying these factors and improving them, the efficiency of the incident command system can be improved.

The hospital incident command system has been recognized as a vital tool for meeting the compliance with accreditation requirements [8] The hospital incident command system also provides opportunities for quantitative and targeted structural assessment [51], assessment of hospital preparedness, processes, identifying, locating and recovering errors [41], exercises for preparedness against disasters [51], and finally creates an assessment system [12]. Also, in evaluating the system, all the levels and functions of the system [51], including the administrative and executive support, planning and adaptation, communication, decision-making, exercises, training and retraining [9] in a real or simulated disaster to be quantitatively and accurately analyzed [23]. Assessing the functions of the hospital incident command system is essential and useful, for example, the effectiveness of the coordination function of the system can be assessed by examining the implementation of cross-measures in accordance with the instructions of the mutual interlocutors [35] and reviewing the coordination with local and foreign authorities [9].

**Table1:** Papers analyzed for the systematic review of literature

**Author**	**Country**	**Year**	**Category**	**Methodology**	**Objective**	**Location of system use**	**Data source**
Howard Backer	USA	2014	Guidebook	reviewing qualitative studies	Updating the Incident Command System Guide	hospitals -health CentersEmergency departments in the hospitals at National and local levels	
Louis N. Molino,Sr	Canada	2006	Book	reviewing qualitative studies	This book points to the applicability of this system for the management of hospitals at the incident scene , and even to the use of it for incidents involving a large number of patients with trauma -induced stress.	hospitals	
P. A. O’ Neill	USA	2005	Original article	reviewing of qualitative texts, results of hospital teachings	Familiarizing surgeons with some principles of responding to incidents or high casualties, including the structure and principles of Hospital Incident Command System	health care facilities hospitals	teachings
Jamal Akhavan Moghaddam	Iran	2006	Original article	reviewing of qualitative texts	Familiarity with Hospital Accident Command System and its implementation	hospitals	hospitals experiences
Lida Shams	Iran	2011	Original article	reviewing of qualitative texts	Identification of Isfahan University of Medical Sciences Hospitals Preparedness for Establishing a Hospital Incident Command System	hospital	Semi-structured interview
ItaloSubbarao	USA	2011	Letter to editor	reviewing of qualitative texts	Appropriate Patient Triage Using Emergency Command System and Deploying Operations center in health emergency situations	Health EmergencyOperations Center	**-**
Takashi Ukai,	Japan	2005	Letter to editor	reviewing of qualitative texts	Evaluation of the effectiveness of Hospital Accident Command System	hospital	**-**
Min Xu	China	2015	original research	literature review	Performance Evaluation of the Public Health Emergency Operations Center	health emergencyoperations center	Texts
Lisa Schoenthal	USA	2015	thesis	case study	Identification of Factors Affecting the Success of the Hospital Incident Command System* (*HICS)	hospital	modeling
Eleanor H. Adams,	USA	2010	Original Article	case study	Investigating the use of Incident Command System for public health threats	health care system	teachings materials
Peter Aitken	Australia	2012	original research	National survey	Application of the incident command system in DTM teams	DMAT(disaster medical assistance teams)	Mail survey
Ali S. Al-Shareef	Saudi Arabia	2017	original research	Cross sectional	Assessing hospital preparedness against disasters	Hospital	collected data
Jeffrey L. Arnold	USA	2005	theoretical discussion	Literature review	Implementation of corrective actions plan of hospital emergency command system in order to adapt hospital emergency management to needs	Hospital	interview
Simon A. Andrew	USA	2012	Original Article	review	Assessment of the problems in providing mental health services in disaster, especially the incident command system	Behavioral and Psychological Services	texts
Karyn Jester Ayers	USA	2013	thesis	qualitative phenomenological	Assessing the roles and capabilities of hospitals during a catastrophic response (disaster)	Hospital	interview
Pamela Autrey	USA	2006	Original Article	qualitative	Investigating the Effect of Knowledge of Highly Valid Location and Teams on Implementing the Incident Command System	Hospital	exercise and interview
Susan Miller Briggs	USA	2009	review article	Literature review	Exploring the principles of the incident command system	Firefighting centers - police and hospitals	**-**
Frederick M. Burkle Jr	USA	2007	Original Article	peer-reviewed literature	Investigating the structure and functions of the incident command system needed for decision making in biological events	HEOC	**-**
Robert K. Kanter	USA	2011	Book (chapter 18)	**-**	Principles used for responding to public health threats by pediatricians	Hospitals - ICU	**-**
Christopher T. Born	USA	2007	Original Article	**-**	Better response to disasters and high-casualties incidents with an emphasis on hospital management system.	Hospitals	**-**
Stephen S. Morse	USA	2006	BOOK(chapter 13)	**-**	Disaster preparedness at health centers and emergency rooms and hospitals	Health Centers - Emergency departments - Hospital	**-**
AhmadrezaDjalali	Iran	2016	Original Article	Qualitative	Personnel competencies required to respond to CBRN incidents and their training needs	Hospital	Delphi
Mohammad Hossein Yarmohammadian	Iran	2011	Educational Research Article	Qualitative	Examining the Challenges, Opportunities and Strategies of the Emergency Hospital Command System (HEICS) for hospital readiness	Hospital	semi-structured interview
Christopher T. Born	USA	2011	Instructional Course Lectures	-	Assessing the importance of the existence of an incident command system in orthopedic surgeons in order to manage disasters in response to disasters	Hospital (surgeons)	-
Saleh Fares	Dubai	2014	Original Article	Review study- instrumentation	Analyzing Hospital Preparedness Levels against Disasters Using HVA Tool	Hospital	Texts - Examining Hospital Experience
Nidaa A. Bajow	Saudi Arabia	2014	Original Article	Cross sectional	Assessing hospital preparedness against disasters	Hospital	Questionnaires collected from hospitals
Pam Hoffner	USA	2009	Original Article	Cross sectional	Application of hospital accident command system in physicians with different specializations	Hospital	modeling
Ahmadreza Jalali	Iran	2012	Original Article	observational study	Measuring the decision-making function using the task descriptions of hospital accident command system	Hospital	Orbital Maneuvering
Dick A. Buck	USA	2006	Original Article	literature review	Conclusion on the use of Hospital Accident command System as an Organizational Management Tool at Disaster Time	Labor Organizations - Public Health - Fire Department - Law Enforcement Agency	Several sources of information related to the nine different incidents
Rouhollah Zaboli	Iran	2014	Original Article	mixed qualitative and quantitative approach	Assessing hospital preparedness	Hospital	Collected questionnaires from hospitals and group discussions
David A. Bradt	Australia	2003	Original Article	Case study	Settlement management and health issues in the recovery and disaster incident rescue phase	health care centers	-
Amy H. Kaji	USA	2006	Original Article	descriptive, cross-sectional survey	Assessing hospital preparedness	Hospital	Questionnaires collected from hospitals
Jessica Jensen	USA	2016	literature review	literature review	knowledge system and the direction of future research	Hospital - fire department and others	literature review
Donald Londorf	USA	1995	special report		Application of Hospital Accident command System	Hospital	**-**
Hesam Seyedin	Iran	2013	original research	qualitative study	Assessing of the effects of major accidents on the preparedness of health organizations in future disasters	Health organizations	semi structured interview
Wendin M Gulbransen	USA	1997	thesis	Cross sectional literature review	Mobile application in the Hospital Accident command System of various disaster phases	Health systems	Texts - teamwork assessment
George U. Njoku	USA	2015	thesis	quantitative design and used survey approach	Studying the compliance of hospitals with the implementation of the components of the National Accident Command System (Incident Command System)Hospital (as one of the ways to estimate hospital preparedness	Hospitals and health systems	Collecting online information from hospitals
Allison T. Chamberlain	USA	2012	Original Article	qualitative study	Reviewing the experiences of H1N1 flu immunization program managers in the United States	Immunization plan	electronic survey
Robert Powers	USA	2007	Feature Article	case study	Description of the successful integration of the principles of incident command in the multi-hospital disinfection program	Multi-hospital disinfection plan	Teamwork and practice
Robert W. Rendin	USA	2005	Original Article	case study	Reviewing the implementation of comprehensive tuberculosis screening programs in health care units based on the principles of the system Incident Command	Health care units	Data on the implementation of the screening plan
Rune Rimstad	Norway	2015	comprehensive review	systematic literature review	Focusing on the commanders at the scene in emergency pre-hospital services with High casualties	Pre-hospital emergency department	Pretext
Marsha Fishbane	USA	2012	supplement article	case study	Use of the Incident Command System in Influenza Vaccination Clinics in Populated classes	Vaccination clinic	teachings
Carl H. Schultz	USA	1996	review articles	literature review	Medical response to sudden deaths after an earthquake	Health care Centers	texts
Tamara L. Thomas	USA	2004	original research	survey	Assessment of hospital training based on the incident command system	hospital	Information obtained from the questionnaire after the exercise
Ernest Sternberg	USA	2004	special report		A Searching for methods and planning terminologies in hospital incidents to promote resilience	hospital	texts
Ahmadreza Jalali	Iran	2015	Research Article	Qualitative	Changes to improve the performance of the incident command system in 2006 version	hospital	Delphi method
Nathan L. Timm	USA	2011	original article	Cross sectional	Describing lessons learned from the 5 years of using the Hospital Command System at the Children's Hospital	hospital	Teachings
Ming-Che Tsai	USA	2004	original article	Quantitative-survey	Assessing the efficiency of the Hospital Accident Command System during an outbreak of Severe acute respiratory syndrome *(*SARS*)* disease in Taiwan in early 2003	hospital	Information obtained from the questionnaire by interview
Chau H. Vu	Taiwan	2012	Clinical Review	case study	This article provides basic information on the general structure of hospital emergency preparedness and specific aspects	hospital emergency department	Teachings
Xin Yantao	China	2010	original research	observational, cross-sectional survey	Hospital Emergency Command Assessment	hospital	self-administered questionnaire
Shahin Shooshtari	Iran	2017	Review Article	review study	Examining the Benefits, Obstacles and Constraints of Using HICS in the Hospital	hospital	texts
Mohammad Hossein Yarmohammadian	Iran	2013	Letter	-	Establishment of Hospital Incident Command System as one of the requirements for better response to incident	hospital	**-**

**Table 2:** Categorization of final articles based on features, strengths, weaknesses, enhancing performance factors, decreasing performance factors, and important factors in assessing the Hospital Accident Command System

**Domain**		**Sub category**	**Variable**
**System features that help to succeed**		Structure	Command- Inter-organizational command- Bureaucratic- Based on military principles- Hierarchical- Rational framework- A distinct chain of Organized command
		Language	Common lexical and linguistic structure- simple- common- common language
		Flexibility and compatibility	Modular design- flexible- Compatible- comparable- Adapted to a variety of events- Adaptation to program events- Scheduled and not-Adhere to the management structure in Changing environments
		Approach	All hazards from top to bottom- Predetermined calling mechanism- Multiple protocols for response
		Application at various levels	Global and international- local- National- daily activities
		Having a command area and control	Specific command area- Appropriate Size Control- Specific monitoring area- Predictable Chain Leadership- Clarity in monitoring
		Providing an appropriate response	Fitted the size of the hospital- Proportional to the extent of the incident- Assigning individuals based on the extent and magnitude of the incident- Activating the sections according to the type and size of the incident
		Management style	Based on precise and extensive goals- Centralized- Defining interactive management components and disaster management structure- Standard System Management Tool- Predictable Management Chain
		Transparency and appropriateness of duties and responsibilities	Posts- responsibilities- Duties Roles- Managerial tasks- Job Descriptions _ Individual- Description of the tasks of external organizations- Task description sheets
		Performance style	The Emergency Response technique at disaster time is not based on a real scenario
		Simplicity	Be simple
		Counting feature	Positions Team performance
		Dependency	Components of response
		Coloration	Specific coloration
**Strengths of system use**		Improved coordination	Operational-Organizational- Inter-organizational- Independent groups- Response actions- Teamwork
		Improved response	Standard response- Facilitating the response- Structured and organized response- Effective- Fast- successful- Effective and efficient- Increased effectiveness- Empowerment- A powerful framework for responding- Proper operation- Resilience promotion
		Resources and facilities	Provision of facilities- Providing enough medical personnel- Effective use of resource- Employing regular human resources- Sharing resources
		improved management	Valid management protocol- Enhancing managerial empowerment - Comprehensive crisis management strategy
		Preparedness status	Preparedness items- Increased hospital readiness- Increased human resource preparedness
		Assessment	Quantitative and targeted structural Assessment of disaster relief- Assessment tool- Assessment system
		Discipline	Reduced chaos caused by disaster- Reduced disruption of decision making
		Planning	Improved planning
		Costs and resources	Reduced costs- Cost Stability- Documentation of costs and resources- Reduced financial losses and injuries -Effective use of resources
		Communications	Providing communication system- Quick communication- Easy connection- Promotion of administrative communications- Preventing unnecessary communications for communication- Effective communication plan
		Organizational capability	In achieving multiple goals
		Reporting and Information	Facilitating information gathering- Facilitating reporting- Information acquisition- Information dissemination- Shared information
		Service delivery	Saving time- Improving the quality of services- Continuity of service- Provide expansion of services
		Patients	Improve the care- Treatment- Triage- Maintaining security
		Personnel	Security- Increased efficiency- increasing the self confidence- team encouragement
**Weakness**		lack of efficiency	in big-complex incidents
		Structure	Unknown military structure
		lack of duplicate and reassure	Executive- Educational- Coordination
		Working with system	complicated health systems
		Inefficient response and confusion	Job description- Ownership responsibilities
		inefficient sharing	between partner organizations
		failure	in health organizations
		System Language	Lack of familiarity with personnel
		Inactivation of system	by the leader despite training
		scope of job descriptions	Wideness
**Factors affecting in increasing efficiency**		Understanding the system	Promoting understanding- Understanding and conceptualization- Knowledge Improving the advanced skills of the system- Promoting an acquaintance culture
		Training and retraining	Staff- Managers-Development of educational materials
		practice	Planning to practice- implementation
		Commitment to implement the system	Organizational Commitment Leadership and Leadership Commitment- Staff Commitment
		coordination	regional
		Financing	Removing Financial Barriers- Allocating funds- Providing Purchase opportunities
		Updating and improved compliance	Plans-Policies- Practices-educational packages- Structures-activities- Processes-Executive Boundaries Compliance with New Threats - New Technologies -Adaptation of the planning stage with the response
		Assessing the Challenges	Disaster program before response
		Facilitating procedures	Removing complex administrative procedures
		Advanced Communications	Use of state of art technologies
		Improved Command Structure and Managers	Appointment of competent managers- Leadership eligibility- Compilation of Command Description-Holding the Committee of Directors-Remove Daily Anxieties
		Compilation and adherence to the rules	Design instructions-Follow the rules-Compliance with the principles of the system
**Points to be considered in the organization's assessment**		Measurement of system functions	Coordination functions-Command-Control-Decision making-System performance-Quantitative analysis
		Assessing the administrative departments of the system	Administrative-executive- Communication-Planning- Adaptation-coordination- Levels of command and personnel -Activating trainings-Comparative time intervals in triage-treatment - transportation - holding meetings, debriefing
**Factors decreasing system efficiency**	**internal barriers**	Cultural	Lack of cultural management-Organizational Culture
		Lack of assessment method	lack of a general method for assessing HICS - lack of a methodology for assessing health-based trainings
		Problems related to managers	Lack of need - support - commitment and belief in the system-Not eligible-Lack of shared management language
		Legal barriers	Lack of legal requirements-Change in the rules and the lack of unity in the command
		Decision making	large number of decision makers
		Financial barriers	High expenditure
		System incompatibility	Incompatibility with existing structures in the hospital
		Lack of a comprehensive plan	Response to hospital disasters and hospital headquarters
		poor communication and coordination	External and internal team communication and coordination
		Lack of competitive space	Development- Planning
	**External barrier**	Parallel work of accountable organizations	Internal- External

**Table 3:** Quality assessment based on the number of subgroups and main groups cited by the authors of the article

**Total**	**The number of main groups referenced**	**Number of subgroups mentioned in the main group of variables and important factors in assessing system performance**	**Number of subgroups mentioned in the main group of factors that reduce the effectiveness of system**	**Number of subgroups mentioned in the main group of factors influencing the system's performance improvement**	**Number of subgroups mentioned in the main group of weaknesses of the system**	**Number of subgroups mentioned in the main group of** **strengths of the system**	**Number of subgroups mentioned in the main group of hospital incident command system features**	**First Author**
15	2	0	0	0	0	6	7	Backer H
13	2	0	0	0	0	6	5	Molino Sr LN
14	2	0	0	0	0	5	7	O'Neill PA
18	3	0	0	2	0	6	7	Akhavan Moghaddam J
15	3	0	0	2	0	4	6	Shams l
2	1	0	0	0	0	1	0	Subbarao I
8	3	0	2	0	0	2	1	TakashiUkai M
6	2	0	0	0	0	1	3	Xu M
19	5	2	1	4	0	1	6	Schoenthal L
7	2	0	0	0	0	4	1	Adams EH
7	2	0	0	0	0	4	1	Aitken P
4	2	0	0	0	0	1	1	Al-Shareef AS
3	1	0	0	2	0	0	0	Arnold JL
7	3	0	0	2	1	1	0	Andrew SA
4	2	0	0	0	0	1	1	Ayers KJ
7	2	0	0	3	0	2	0	Autrey P
8	3	0	0	2	0	1	2	Briggs SM
4	2	0	0	1	1	0	0	Burkle FM
7	2	0	0	0	0	4	1	Kanter RK
10	3	0	0	1	0	2	4	Born CT
10	3	0	0	0	2	1	4	Morse S
5	2	0	0	0	0	2	1	Djalali A (2016)
24	4	7	0	4	0	4	5	Yarmohammadian MH (2011)
7	2	0	0	0	0	2	3	Born CT
8	2	0	0	0	0	1	5	Fares S
5	2	0	0	0	0	1	2	Bajow NA
4	1	0	0	0	0	3	0	Hoffner P
7	3	2	1	1	0	0	0	Djalali A (2012)
5	2	0	0	0	1	3	0	Buck DA
3	1	0	0	0	0	0	2	Zaboli R
3	1	0	0	0	0	2	0	Bradt DA
3	1	2	0	0	0	0	0	Kaji AH
6	2	0	0	2	0	-	2	Jensen J
9	3	1	0	0	0	2	3	Londorf D
3	1	0	0	2	0	0	0	Seyedin H
4	1	0	0	0	0	0	3	Gulbransen WM
4	2	0	0	0	0	1	1	Njoku GU
4	1	0	0	0	0	3	0	Chamberlain AT
5	2	0	0	0	0	2	1	Powers R
8	2	0	0	0	0	3	3	Rendin RW
6	3	0	0	1	1	0	1	Rimstad R
8	2	0	0	0	0	1	5	Fishbane M
5	2	0	0	2	0	0	1	Schultz CH
6	2	0	2	0	0	2	0	Thomas TL
7	2	0	0	0	0	4	1	Sternberg E
6	2	1	0	3	0	0	0	Djalali A (2015)
8	2	0	0	1	5	0	0	Timm NL
5	2	0	0	1	0	0	2	Tsai M-C
7	3	0	0	1	0	1	2	Vu CH
3	1	0	0	0	0	2	0	Yantao X
13	2	0	0	0	0	6	5	Shooshtari S
14	3	0	0	7	0	3	1	Yarmohammadian MH (2013)

## Conclusion

Hospital incident command system is one of the hospital's essential requirements for coping, respond and managing emergencies and disasters. The condition of applying and improving the efficiency of this system is to recognize the principles, characteristics, strengths and weakness of it by hospital staff and managers. It is also necessary to assess and evaluate the performance of the system and its functions with a scientifically valid method. Continuous assessment and recognition of the problems and strengths of the system will improve its efficiency. Therefore, hospital managers and health decision-makers need to plan and done the HICS's assessment, identify its strengths and problems, and train its principles and characteristics for hospital settings.

## Conflict of Interest:

None declared.
